# Systemic Lupus Erythematosus With Isolated Psychiatric Symptoms and Antinuclear Antibody Detection in the Cerebrospinal Fluid

**DOI:** 10.3389/fpsyt.2019.00226

**Published:** 2019-04-25

**Authors:** Eva M. Lüngen, Viktoria Maier, Nils Venhoff, Ulrich Salzer, Rick Dersch, Benjamin Berger, Anne N. Riering, Kathrin Nickel, Bernd L. Fiebich, Patrick Süß, Simon J. Maier, Karl Egger, Ludger Tebartz van Elst, Dominique Endres

**Affiliations:** ^1^Section for Experimental Neuropsychiatry, Department of Psychiatry and Psychotherapy, Medical Center–University of Freiburg, Faculty of Medicine, University of Freiburg, Freiburg, Germany; ^2^Department of Psychiatry and Psychotherapy, Medical Center–University of Freiburg, Faculty of Medicine, University of Freiburg, Freiburg, Germany; ^3^Department of Rheumatology and Clinical Immunology, Medical Center–University of Freiburg, Faculty of Medicine, University of Freiburg, Freiburg, Germany; ^4^Department of Neurology, Medical Center–University of Freiburg, Faculty of Medicine, University of Freiburg, Freiburg, Germany; ^5^Department of Neuroradiology, Medical Center–University of Freiburg, Faculty of Medicine, University of Freiburg, Freiburg, Germany

**Keywords:** systemic lupus erythematosus, neuropsychiatric systemic lupus erythematosus, schizophrenia, obsessive-compulsive disorder (OCD), psychosis

## Abstract

**Background:** Organic psychiatric disorders can be caused by immunological disorders, such as autoimmune encephalitis or systemic lupus erythematosus (SLE). SLE can affect most organs, as well as the central nervous system (CNS). In this paper, we describe a patient with an isolated psychiatric syndrome in the context of SLE and discuss the role of antibody detection in the cerebrospinal fluid (CSF).

**Case presentation:** The 22-year-old German male high school graduate presented with obsessive–compulsive and schizophreniform symptoms. He first experienced obsessive–compulsive symptoms at the age of 14. At the age of 19, his obsessive thoughts, hallucinations, diffuse anxiety, depressed mood, severe dizziness, and suicidal ideation became severe and did not respond to neuroleptic or antidepressant treatment. Due to increased antinuclear antibodies (ANAs) with anti-nucleosome specificity in serum and CSF, complement activation, multiple bilateral white matter lesions, and inflammatory CSF alterations, we classified the complex syndrome as an isolated psychiatric variant of SLE. Immunosuppressive treatment with two times high-dose steroids, methotrexate, and hydroxychloroquine led to a slow but convincing improvement.

**Conclusion:** Some patients with psychiatric syndromes and increased ANA titers may suffer from psychiatric variants of SLE, even if the American College of Rheumatology criteria for SLE are not met. Whether the psychiatric symptoms in our patient represent a prodromal stage with the later manifestation of full-blown SLE or a subtype of SLE with isolated CNS involvement remains unclear. Regardless, early diagnosis and initiation of immunosuppressive treatment are essential steps in preventing further disease progression and organ damage. Intrathecal ANAs with extractable nuclear antigen differentiation may be a more sensitive marker of CNS involvement compared with serum analyses alone.

## Background

Organic psychiatric disorders might be of immunological, infectious, epileptic, neurodegenerative, traumatic, metabolic, or vascular origins (1, 2). In recent years, limbic or nonlimbic autoimmune encephalitis received increased interest because each can mimic primary psychiatric and neuropsychiatric disorders (2–4). Most of these disorders are associated with autoantibodies (abs) directed against antigens on the cell surface or in the intracellular compartment (5). Hashimoto thyroiditis and rheumatic disorders, such as systemic lupus erythematosus (SLE), can also be associated with psychiatric involvement that allows for successful immunomodulatory treatment approaches (6–9).

SLE is a prototypic systemic autoimmune disease that can affect the central nervous system (CNS) as well as the rest of the body, including joints, skin, kidneys, heart, lungs, blood vessels, or the hematopoietic system. The typical age for mostly women to become ill is between 16 and 55 years (10); the peak age in females is between 45 and 69 years, while that in males is between 40 and 89 years (11). SLE is characterized by the presence of antinuclear abs (ANAs); these abs can affect different cell types, which explains the diversity of symptoms. The reference standard for the diagnosis of this extremely heterogeneous multisystemic disease is still clinical diagnosis by an SLE expert. The main reason for the use of SLE classification criteria is to ensure a consistent definition of SLE, especially for clinical trials and surveillance. The most commonly used criteria are those established by the American College of Rheumatology (ACR) in 1982 and revised in 1997 (12, 13; https://www.rheumatology.org/Practice-Quality/Clinical-Support/Criteria/ACR-Endorsed-Criteria; see **Box 1A**). If 4 or more of the 11 criteria, including at least 1 immunological and 1 clinical criterion, are present simultaneously or serially at any point in time, SLE should be highly suspected. Although the ACR’s 1997 criteria have generally performed well, concerns have been raised regarding the limited sensitivity of these criteria. Neuropsychiatric conditions, for example, might be underrepresented in the criteria. Up to 75% develop neuropsychiatric symptoms, such as cognitive dysfunction, seizures, and other psychiatric syndromes, such as mood disorders, anxiety, or psychosis (14). A subgroup of SLE patients mainly presents with manifestations involving the central, peripheral, or autonomic nervous system. These are referred to as neuropsychiatric SLE (NPSLE) (15). In 1999, the ACR published 19 case definitions of NPSLE to facilitate diagnosis and ensure comparability across clinical studies. Twelve definitions described syndromes associated with CNS involvement (15) (**Box 1B**).

Box 1 **Criteria for (neuropsychiatric) systemic lupus erythematosus**

**A: Criteria for systemic lupus erythematosus of the American College of Rheumatology** (12,13; https://www.rheumatology.org/Practice-Quality/Clinical-Support/Criteria/ACR-Endorsed-Criteria)

1. Malar rash; 2. discoid rash; 3. photosensitivity; 4. oral ulcers; 5. arthritis; 6. serositis; 7. renal involvement; 8. neurological involvement (seizures or psychosis); 9. hematological involvement (hemolytic anemia, leucopenia, lymphopenia, thrombocytopenia); 10. immunological changes (anti-dsDNA antibodies, anti-Sm antibodies, antiphospholipid antibodies, and 11. antinuclear antibodies.

**B: Case definitions of neuropsychiatric systemic lupus erythematosus** (15):

1. Aseptic meningitis; 2. cerebrovascular disease; 3. demyelinating syndrome; 4. headaches (migraines and benign intracranial hypertension); 5. movement disorders (chorea); 6. myelopathy; 7. seizure disorders; 8. acute confusional states; 9. anxiety disorders; 10. cognitive dysfunction; 11. mood disorders; and 12. psychosis.

Two distinct pathogenic mechanisms, tissue inflammation and thrombotic–ischemic events, have been recognized thus far. Increased ANA serum titers were detected in >95% of patients (16); however, older studies showed even lower ANA positivity (17). Positive dsDNA abs were found at a rate of 37–80% (16), and they were interpreted as a predictor of disease exacerbation (18). The metanalytic data showed that anti-nucleosome abs have comparable specificity but higher sensitivity than anti-dsDNA abs do for the diagnosis of SLE (19). Cerebrospinal fluid (CSF) pleocytosis was found in 30%, and oligoclonal bands (OCBs) were detected in 25–42% of cases (20). In the cerebral magnetic resonance imaging (cMRI), small focal hyperintensities, mainly subcortical frontoparietal or periventricular, can be detected in 15–60% of the patients. Electroencephalography (EEG) generally shows unspecific slowing (21). NPSLE treatment can be implemented with corticosteroids alone or in combination with other immunosuppressive drugs, including cyclophosphamide for remission induction or azathioprine for maintenance therapy (22). Antimalarial drugs (e.g., hydroxychloroquine) have been suggested for the prevention of NPSLE in SLE patients (23).

## Case Presentation

We present the case of a 22-year-old German male high school graduate with a complex psychiatric syndrome including obsessive–compulsive, schizophreniform, and derealization phenotypes. In May 2016, at age 19, there was a sudden exacerbation of these syndromes. At age 14, he first experienced obsessive–compulsive symptoms (i.e., obsessive aggressive thoughts and compulsive avoidance acts). However, he recognized that the obsessional thoughts were a product of his own mind, and these symptoms were well compensated for at the time, enabling him to live a mostly normal life. After his final examinations at school, he consumed cannabis five times. He then experienced an exacerbation of his obsessive–compulsive symptoms and suffered from more severe obsessional thoughts, including the idea that he could injure other people and himself. Furthermore, he experienced involuntary obscene thoughts. At the time, he fought such thoughts and continued to recognize that the obsessional thoughts and impulses were a product of his own mind. He also suffered from hallucinatory symptoms, such as auditory hallucinations (i.e., hearing voices) and optical distortions (i.e., the shape of leaves on the ground appearing distorted). He developed diffuse anxiety and agitation and described extreme dizziness, as if he had drunk “five beers.” Because of his depressed mood and obsessive–compulsive symptoms, he experienced suicidal ideation and complained of difficulty falling asleep and reduced energy levels, especially in the morning.

**Figure 1:**
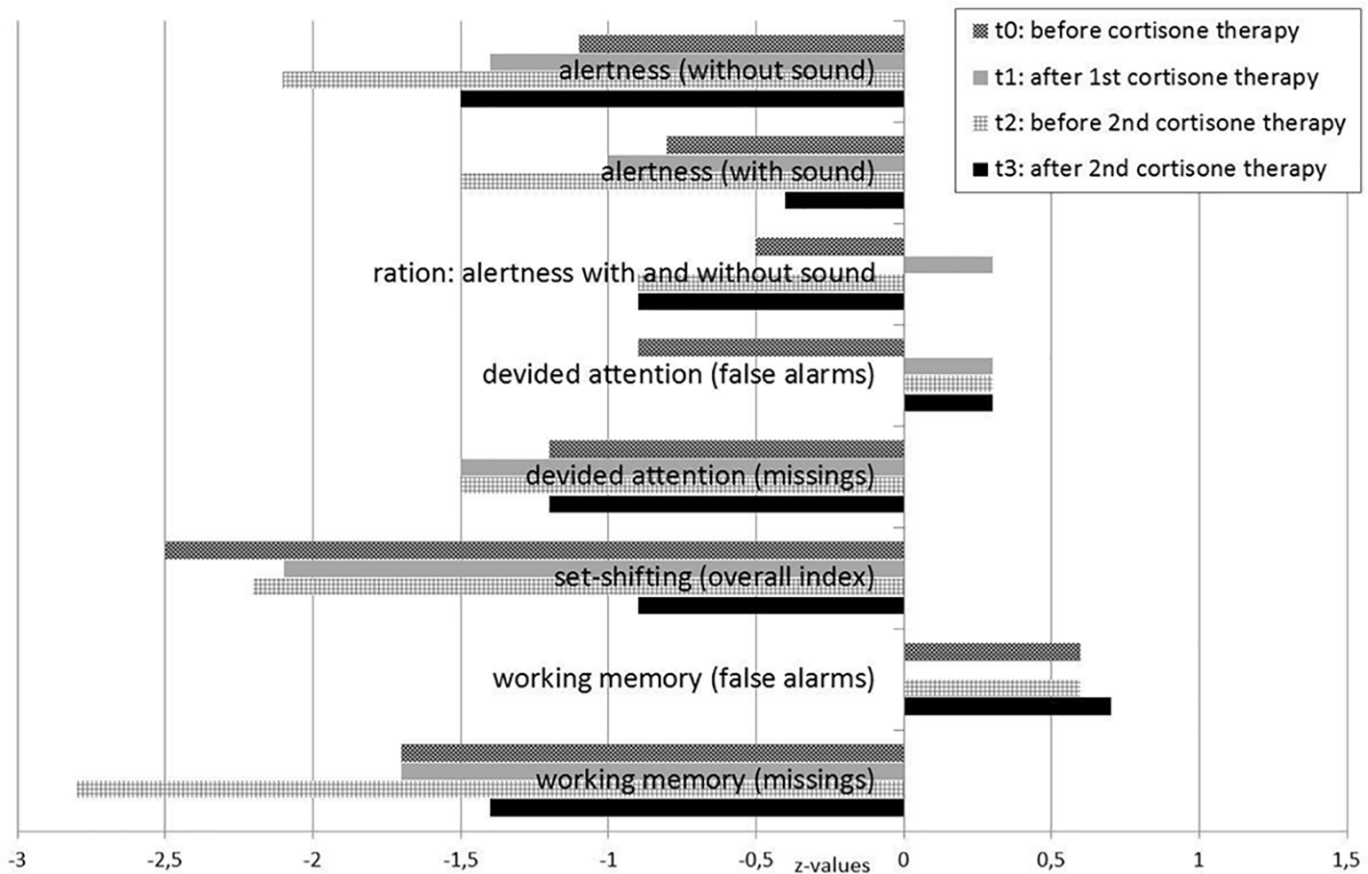
Test for attentional performance during the course of the disease.

Due to the severity of these symptoms, he was first hospitalized at age 19. He received pharmacological treatment with selective serotonin reuptake inhibitors for the obsessive–compulsive symptoms (citalopram up to 40 mg/day), neuroleptics for the schizophreniform symptoms (olanzapine up to 20 mg/day, risperidone up to 5 mg/day, and aripiprazole up to 7.5 mg/day; higher doses led to an increase in inner restlessness), carbamazepine for neuronal network stabilization up to 500 mg/day, and an anticholinergic agent because of extrapyramidal side effects (biperiden up to 4 mg/day) over a period of 7 months without significant improvement. Lorazepam (up to 2 mg/day) led to a transient reduction in anxiety. Seven months after symptom onset (December 2016, under treatment with aripiprazole 7.5 mg/day and carbamazepine 500 mg/day), his mental state still revealed obsessive thoughts, depressed mood, and diffuse anxiety. Moreover, he suffered from attention and concentration deficits (**Figure 1**, t0), inner restlessness, signs of derealization (with altered, slowed, and delayed perception of his environment), and severe dizziness (still comparable with having drunk five beers). The obsessive–compulsive thoughts were still present and very difficult to manage.

### Developmental, Somatic, and Family History

There were no *in utero* or birth complications, febrile convulsions, seizures, inflammatory brain diseases, or cerebral contusions in the patient’s history. When entering primary school, he showed subsyndromal symptoms of inattention and motor hyperactivity. Nevertheless, he finished high school successfully and his further somatic history was unremarkable. He occasionally consumed alcohol and illegal drugs (nitrous oxide three times and cannabis five times), but there was no history of severe substance abuse. The family history showed that his grandmother suffered from depression, and his mother was diagnosed with insulin-dependent diabetes mellitus. There were no known rheumatic diseases in the family history.

### Investigations

The neurological examination was normal throughout the course of the disease. Initially, the CSF analyses (3 months after exacerbation, August 2016) showed positive CSF-specific OCBs. Five months after the first steroid pulse treatment (December 2016), the patient’s state deteriorated (May 2017). At that time, CSF analysis showed a mild pleocytosis (white blood cell count = 14/µl; reference <5/µl). The initial immunological screening 6 months after exacerbation in November 2016 revealed only a weak positive ANA in the indirect immunofluorescence assay. Another 6 months later (1 year after exacerbation, May 2017), we found clearly increased ANA titers in both serum and CSF (serum: titer = 800 IU; CSF: titer <100 IU) with anti-nucleosome specificity, which was also detectable in serum and CSF. At that time, we also detected decreased levels of complement component C4 and slightly increased C3d serum concentrations as indicators for increased complement activation.

**Figure 2:**
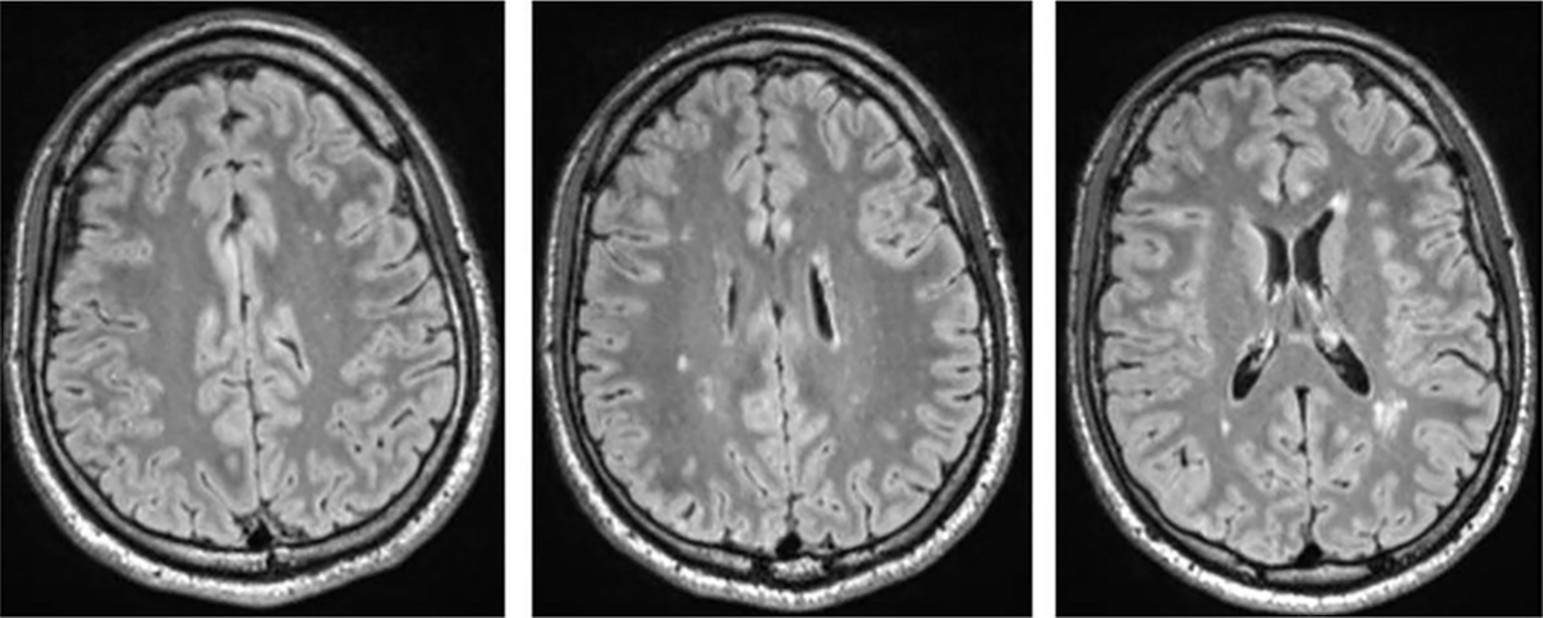
T2w fluid-attenuated inversion recovery (FLAIR) cerebral magnetic resonance imaging (cMRI) shows multiple disseminated dotted bilateral periventricular to subcortical white matter lesions. Shown are images of the first cMRI performed 6 months after symptom exacerbation in November 2016.

Testing for rheumatoid factors, antiphospholipid abs, lupus anticoagulant, antineutrophil cytoplasmic abs, and a broad set of antineuronal and anti-thyroid abs was negative. In the cMRI, multiple diffuse periventricular white matter lesions were apparent in repeated examinations throughout the course (**Figure 2**). The lesions were stable. Furthermore, there was a slightly enlarged adenohypophysis not yet affecting the chiasma opticum. The hormone screening did not detect any pathological hormone activity. The fluorodeoxyglucose positron emission tomography was normal. Repeated EEGs exhibited intermittent slowing (**Table 1**). The neuropsychological test of attentional performances showed severe deficits in alertness, divided attention, set shifting, and working memory (**Figure 1**, t0). There were no further clinical, systemic SLE signs such as skin or inner organ involvement.

**Table 1:** Overview of diagnostic findings.

**Serum basic diagnostics and blood count**	**Repeatedly normal renal, and thyroid values.** Repeatedly no relevant alterations in peripheral differential blood cell count were observed (except twice increased leucocyte count during steroid treatment). No lymphocytopenia was found.
**Rheumatological testing**	11/2016 (6 months after symptom exacerbation): **Immunofluorescence test for antinuclear abs (ANAs) was weakly positive.** Normal values for CH50 (*89%, reference area 65–115%*), C3 (*1.24 g/L; reference area 0.90–1.80 g/L*), C4 (*0.23 g/L; reference area 0.10–0.40 g/L*), C3d (*8.40 mg/L; reference value <9 mg/L*). Antineutrophil cytoplasmic (ANCA) and anti-β2 glycoprotein I IgM and IgG antibodies were not increased (APAs); anti-dsDNA and anti-SM abs were negative. 05/2017 (12 months after first exacerbation): **Increased ANAs with anti-nucleosome specificity** (*serum: titer of 1:800; CSF: titer <1:100; reference value <1:50*). **Slightly increased C3d concentration** (*12.90 mg/L; reference value <9 mg/L*). **Slightly decreased C4** (*0.09 g/L; reference area 0.10–0.40 g/L*). ANCA, APAs, anti-dsDNA, and anti-SM abs were still negative. The lupus anticoagulant test was negative. 01/2019 (32 months after first exacerbation): Anti-ribosomal P protein and anti-C1q antibodies in the serum were negative. Direct Coombs test results were unremarkable.
**Serum autoantibody analyses**	Repeatedly no increased thyroid abs (against thyroglobulin, thyroid peroxidase, and thyroid-stimulating hormone receptor). Repeatedly (08/16; 05/17) no abs against intracellular onconeural or intracellular synaptic antigens (Yo, Ri, Hu, CV2/CRMP5, Ma1/2, SOX1, GAD65, amphiphysin). Aquaporin abs negative (5/2017)
**Urine**	Repeatedly no signs of proteinuria (except slightly increased protein concentration 06/2017 (*17 mg/dL, reference <15 mg/dL*), no detection of red blood cells Normal protein/creatinine ratio (06/2017).
**Cerebrospinal fluid analyses**	8/2016 (3 months after symptom exacerbation): Normal WBC count (2/µl; reference < 5/µl); normal protein concentration: 426 mg/L; *reference <450 mg/L*; normal albumin quotient: 4.7; *age-dependent reference <6.5 × 10^−3^*; **detection of two CSF specific OCBs**, IgG index not increased (*0.57; reference ≤0.7*). Abs against neuronal cell surface antigens [NMDA-R, AMPA-R, GABA-B-R, VGKC-complex (LGI1, Caspr2)] were negative. 05/2017 (12 months after first exacerbation): **increased WBC count** (14/µl; reference <5/µl); **increased protein concentration: 460 mg/L**; *reference <450 mg/L*; normal albumin quotient: 4.5; *age-dependent reference < 6.5×10^−3^*. **Only one CSF specific OCB**, IgG index not increased (0.54; reference ≤0.7). Antibodies against neuronal cell surface antigens (NMDA-R, AMPA-R, GABA-B-R, VGKC-complex [LGI1, Caspr2]) were negative. 7/2017 (14 months after first exacerbation): Normal WBC count (2/µl; reference <5/µl); normal protein concentration: 393 mg/L; *reference <450 mg/L*; normal albumin quotient: 4.3; *age-dependent reference <6.5 × 10^−3^*). **Two CSF specific OCBs**, IgG index not increased (0.59; reference ≤0.7). Negative MRZ reaction.
**Cerebral magnetic resonance imaging** (7/2016, 11/2016, 5/2017, 7/2017)	2, 6, 12, and 14 months after first exacerbation: Repeatedly multiple bilateral diffuse periventricular white matter lesions without contrast enhancement and with stable lesions; no indication for acute ischemia or intracranial hemorrhage; deformed and steadily increasing adenohypophysis not yet affecting the optic chiasm.
**Electroencephalography** (8/2016, 11/2016, 5/2017)	Initially normal alpha EEG (3 months after first exacerbation), in the course of the disease (occipital accentuated), sporadic intermittent rhythmic theta activity (6 months after first exacerbation) and repeated slow delta activity, partly with generalization (12 months after first exacerbation). No epileptic discharges.
**Electrophysiological testing** (8/2016)	3 months after first exacerbation: Somatosensory evoked potentials of the median nerve, the ulnar nerve, and the tibial nerve (in each case on both sides) were normal, as well as visual evoked potential responses.
**FDG-PET** (12/2016)	7 months after first exacerbation (WBC count was normal during that time): Age-appropriate utilization of glucose; no characteristic pattern of a neurodegenerative or inflammatory central nervous system disease.
**Hormone screening** (7/2017, 14 months after first exacerbation, performed because of enlarged adenohypophysis)	In the glucose suppression test, a suppression of GH to values <1 ng/ml was observed over time (suppression works adequately, which speaks against acromegaly). ACTH test was unremarkable (which speaks against adrenocortical insufficiency). No signs of hypopituitarism. No other hormone overproduction (normal prolactin, etc).

Abs, antibodies; ACR, American College of Rheumatology; CSF, cerebrospinal fluid; IU, international unit; Yo, initials of the first patient; Ri, initials of the first patient; Hu, initials of the first patient; CRMP, anti-collapsin response-mediator protein; SOX1, sex-determining region Y (SRY)-box protein 1; GAD, glutamic acid decarboxylase; WBC, white blood cells; OCBs, oligoclonal bands; NMDA-R, N-methyl-d-aspartate receptor; AMPA-R, amino-3-hydroxy-5-methyl-4-isoxazole-propionic acid-receptor; GABA-B-R, gamma-aminobutyric-acid-B-receptor; VGKC voltage-gated potassium channel; LGI1, leucine-rich glioma inactivated-1 protein; Caspr2, contactin-associated protein 2; EEG, electroencephalography; FDG-PET, fluorodeoxyglucose positron emission tomography; GH, growth hormone; ACTH, adrenocorticotropic hormone.

### Differential Diagnostic Considerations

Focusing on the symptoms our patient developed during his youth, a diagnosis of obsessive–compulsive disorder with obsessive thoughts could be considered. The fact that disease symptoms exacerbated shortly after the consumption of cannabis could point to an acute episode of drug-induced psychosis. If the auditory and optical hallucinations together with signs of derealization persisted over time, paranoid hallucinatory schizophrenia could be a plausible classification, though only if all organic signs were considered pathogenetically irrelevant. The initial organic findings (positive CSF-specific OCBs, disseminated white matter lesions, no antineuronal abs, unspecific rheumatological findings) were interpreted as post-inflammatory changes. Finally, the increased ANAs with anti-nucleosome specificity in CSF and serum, serum complement activation, CSF pleocytosis, OCBs, and cognitive and psychiatric disturbances cumulated to the final diagnosis of NPSLE. Although the patient did not completely fulfill the ACR criteria for SLE, the diagnosis was established. Other supporting biomarkers, such as anti-ribosomal P protein and anti-C1q abs, as well as the direct Coombs test, were unremarkable. Autoimmune encephalitis would also be conceivable for differential diagnosis (4). However, the established antineuronal abs were negative in serum and CSF.

### Immunosuppressive Treatment and Course of the Disease

Following initial assessment and based on the therapy resistance and inflammatory CSF changes with CSF-specific OCBs, we performed a high-dose corticosteroid pulse therapy with a daily dose of 1,000 mg methylprednisolone administered intravenously for five consecutive days in the context of continued treatment with aripiprazole and carbamazepine in December 2016, which was 7 months after symptom exacerbation. Following the steroid treatment, the patient’s mood, cognitive deficits, motivation, and dizziness improved. Moreover, his obsessive–compulsive symptoms were reduced. Methylprednisolone was subsequently continued orally and tapered over approximately 5 weeks (37 days). Except for steroid acne, no relevant adverse events became evident. After this immunosuppressive treatment, the patient was able to begin a volunteer social year, and treatment with aripiprazole and carbamazepine was continued. However, he still had obsessive–compulsive symptoms and cognitive deficits and felt dizzy (comparable with having drunk two or three beers). Clinically, we did not believe that these symptoms were side effects of carbamazepine because the symptoms were already reported prior to the treatment with carbamazepine, and blood concentrations were within the reference level. Neuropsychological testing showed no relevant improvement (**Figure 1**, t1). Over the next 4 months, his mental condition worsened again.

In May 2017, 1 year after his initial symptoms exacerbated, we repeated the diagnostic workup. The patient showed mild pleocytosis in the CSF and laboratory findings compatible with SLE (**Table 1**). We performed a second steroid pulse therapy with 500 mg/day methylprednisolone for five consecutive days with oral tapering over 8 weeks. At the same time, carbamazepine was stopped, and aripiprazole was reduced to 5 mg/day. As maintenance treatment, the patient received methotrexate (up to 17.5 mg/week) orally in combination with folic acid. Due to the threat of azoospermia, cyclophosphamide, which normally shows good results for NPSLE, was not initially used. The CSF white blood cell count normalized, and the cMRI showed that the multiple diffuse periventricular to subcortical white matter lesions remained stable. Neuropsychological testing revealed an improvement in alertness, mental flexibility, and working memory [**Figure 1**, t2 (before) versus t3 (after) the second steroid pulse treatment]. Two months later (August 2017, 15 months after his symptoms first exacerbated), we added the antimalarial drug hydroxychloroquine (200 mg/day). The maintenance therapy consisting of methotrexate (17.5 mg/week), hydroxychloroquine (200 mg/day), and aripiprazole (5 mg/day) resulted in a slow but substantial improvement of inner restlessness, cognitive deficits, derealization symptoms, and dizziness. Obsessive–compulsive symptoms disappeared completely. Over several months, he still felt dizzy, like “having drunk one beer.” Sixteen months later (December 2018), the patient was with without dizziness (“zero beers”), aripiprazole treatment was stopped in the meantime, and he enrolled in a vocational training program. Here, he was able to attend vocational school, but on long working days, he cognitively reached his limits.

## Discussion

In this paper, we present the case of a male patient with a severe psychiatric variant of NPSLE suffering from obsessive–compulsive and schizophreniform symptoms who responded slowly but very well to immunological treatment.

### Diagnostic Considerations

Initially, the cMRI and CSF showed signs of inflammatory CNS involvement (i.e., disseminated periventricular white matter lesions and isolated OCBs in the CSF). At that time, the ANA immunofluorescence test was only weakly positive. In view of the organic findings, the other differential diagnostic considerations mentioned above (e.g., drug-induced disorder, obsessive–compulsive disorder, or schizophreniform syndrome) were rejected. Six months after the first assessment, the ANA titers had clearly increased, with anti-nucleosome specificity found in the CSF and serum. Along with the complement alterations, the inflammatory changes in the CSF, the cMRI and EEG alterations, as well as the clinical manifestation with psychosis, we diagnosed NPSLE. Although the ACR 1997 classification criteria for SLE were not completely fulfilled, we deemed it justified to diagnose NPSLE in this patient. The strongest indicators were as follows: A) the presence of ANAs in a young male patient, which is unusual and raises suspicion for SLE; B) the detection of anti-nucleosome abs, which have the same specificity and even higher sensitivity than anti-dsDNA abs for SLE (19); C) complement activation as an indicator for disease activity; and D) the clinical presentation with psychiatric symptoms and corresponding alterations in CSF diagnostics, cMRI, and EEG.

Neuropsychiatric manifestations usually occur early in the course of SLE, and in some patients, neuropsychiatric symptoms remain the only clinical manifestations of SLE. This case demonstrates the difference between classification and diagnosis. Classification defines a homogenous group of patients for a research purpose with suboptimal sensitivity, whereas diagnosis focuses on the individual patient’s therapy and prognosis and diagnosis should also lead to the treatment of patients who do not meet the classification due to non-100% sensitivity but who should still receive treatment (24). There are limitations of the most widely used ACR 1997 classification (but not diagnostic) criteria for SLE. While photosensitivity and skin and mucous membrane involvement seems to be overrepresented, other conditions, such as neuropsychiatric involvement, might be underrepresented. Furthermore, important immunological tests, such as complement fractions, anti-b2-glycoprotein, or anti-nucleosome abs, have not been considered thus far. While our patient fulfilled only 2 (i.e., psychosis and the presence of ANA) of the possible 11 ACR criteria, he would have fulfilled 3 (i.e., psychosis, the presence of ANA, and lowered C4 levels) of the recently proposed Systemic Lupus International Collaborating Clinics (SLICC) 2012 SLE classification criteria set (25). According to the SLICC 2012 criteria, the classification of SLE also requires at least four criteria with at least one clinical and one laboratory criteria, but this case is a good example of the relevance of the composition of the applied classification criteria set.

The European League Against Rheumatism and ACR are aware of this situation, and therefore, new SLE classification standards based on weighted criteria and a continuous probability scale are currently being developed. Recent studies investigated whether the detection of certain abs (e.g., increased ANA titers) in combination with symptoms manifested in one organ system (i.e., incomplete clinical pictures of SLE) may point to a prodromal stage of SLE (26, 27). Several papers described cases with initially insufficient numbers of diagnostic findings to fulfill the ACR criteria for SLE, for example, cases with initial manifestations of the gastrointestinal tract that later developed into full-blown SLE (28, 29). There are also many cases of SLE with the presence of ANA and isolated kidney involvement with glomerulonephritis. Despite not fulfilling the ACR classification criteria, the diagnosis can be made in cases of biopsy-proven lupus nephritis. Mack et al. published a case report of a woman who suffered from a wide spectrum of psychiatric symptoms that had relapsed several times over the 25-year disease duration. Throughout the entire period, several findings indicated an immunological process as a cause of her illness, but it took approximately 23 years after first suspecting an autoimmune disorder before the diagnosis of (NP)SLE was made (9). Given this background, we conclude that an isolated psychiatric variant of SLE might well be plausible in our patient. Whether the psychiatric symptoms in our patient represent a prodromal stage with the later manifestation of full-blown SLE or a subtype of SLE with isolated CNS involvement remains unclear. Because of the autoimmune pathogenesis of this systemic disease, the continuation of the immunosuppressive maintenance therapy should prevent further disease progression and organ damage. Nevertheless, even if the patient does not currently fulfill the criteria for SLE, early diagnosis is possible, and initiating immunosuppressive treatment is essential. Therefore, it is important to consider that isolated psychiatric variants of SLE may occur without other organ manifestations.

### Role of Cerebrospinal Fluid Antibody Detection

In our case, we detected ANAs with anti-nucleosome specificity not only in the serum but also in the CSF, which supported the hypothesis that the CNS is affected. Even if the CSF examinations are not established, one could hypothesize that the short-term cannabis use may have led to a temporary blood–brain barrier dysfunction and allowed the ANAs’ passage to the CNS. This could explain the rapid deterioration after cannabis consumption. Besides direct ab effects, increased interferon signaling might lead to reactive microglia and therefore could lead to neuronal damage and loss of synapses (30). Nevertheless, the precise pathophysiology of immunological mechanisms in affected brain tissue and thereby the resulting neuropsychiatric symptoms have not yet been fully clarified (31).

However, if the detection of ANAs in the CSF is a marker of CNS involvement, this would be of immense help in clinical practice. ANAs detected in serum alone are too unspecific to function as a marker for CNS involvement in clinical practice. They were found with similar prevalence rates in patients with schizophrenia and in controls in earlier studies (32). Higher rates of serum ANAs might also be due to drug-induced ANA titers, which are often observed in psychiatric patients (33). Therefore, the serum findings’ significance remains unclear in individual patients with psychiatric disorders. CSF analyses could help diagnose an autoimmune connective tissue disease with CNS involvement when detecting increased intrathecal ANA titers and other inflammatory alterations, such as increased white blood cell counts and CSF-specific OCBs. A metanalysis showed a higher rate of positive titers for antineuronal abs in the CSF of patients with NPSLE compared with SLE patients (34). In classical patients with NPSLE, an intrathecal ab synthesis was previously shown (35). Anti-SSA abs were found in CSF of patients with SLE (36) and Sjögren’s syndrome earlier (37). Future studies should investigate the sensitivity and specificity of intrathecal ANA synthesis with ENA specification in psychiatric variants of SLE.

## Conclusion

Our case clearly illustrates the relevance of this issue for clinical psychiatry. Had we rejected the SLE diagnosis based on the existing classification criteria, we would not have chosen the immune-modulatory treatment approach, which proved to be extremely successful in the long run in our patient. Intrathecal ANAs with extractable nuclear antigen differentiation may be a more sensitive marker of CNS involvement than serological testing alone is.

## Ethics Statement

The patient has given his signed written informed consent for this case report, including the presented images, to be published.

## Author Contributions

DE, LT, NV, and RD treated the patient. EL, VM, and DE performed the data research: EL and DE summarized the case report, and VM performed a literature search. EL, VM, and DE wrote the paper. KE performed the cMRI analyses. RD and BB performed the EEG and CSF analyses and neurological interpretation. NV and US performed the rheumatological analyses, clinical interpretation and therapy suggestion. AR performed the neuropsychological testing. BF, SM, PS, and KN supported the interpretation of diagnostic findings. All authors were critically involved in the theoretical discussion and composition of the manuscript. All authors read and approved the final version of the manuscript.

## Funding

The article processing charge was funded by the German Research Foundation (DFG) and the University of Freiburg in the funding program Open Access Publishing.

## Conflict of Interest Statement

NV: Advisory boards, lectures, research or travel grants within the last three years: Janssen-Cilag, Roche, Novartis, AbbVie, GSK, Medac, Pfizer. BB: Travel grants and/or training expenses from Bayer Vital GmbH, Ipsen Pharma GmbH, Biogen GmbH, Norvartis, and Genzyme, as well as lecture fees from Ipsen Pharma GmbH, Alexion Pharma GmbH, Sanofi Aventis GmbH, Roche Pharma AG and Merck Serono GmbH. LT: Advisory boards, lectures, or travel grants within the last three years: Eli Lilly, Janssen-Cilag, Novartis, Shire, UCB, GSK, Servier, Janssen, and Cyberonics. The remaining authors declare that the research was conducted in the absence of any commercial or financial relationships that could be construed as a potential conflict of interest.
